# Prevalence and Predictors of Video Game Addiction: A Study Based on a National Representative Sample of Gamers

**DOI:** 10.1007/s11469-015-9592-8

**Published:** 2015-09-23

**Authors:** Charlotte Thoresen Wittek, Turi Reiten Finserås, Ståle Pallesen, Rune Aune Mentzoni, Daniel Hanss, Mark D. Griffiths, Helge Molde

**Affiliations:** 1 Department of Clinical Psychology, University of Bergen, Christiesgate 12, 5015 Bergen, Norway; 2 Department of Psychosocial Science, University of Bergen, Bergen, Norway; 3 Faculty of Social and Cultural Sciences and Social Work, Hochschule Darmstadt - University of Applied Sciences, Darmstadt, Germany; 4 International Gaming Research Unit, Psychology Division, Nottingham Trent University, Nottingham, UK

**Keywords:** Video game addiction, Prevalence, Personality traits, Psychosomatic health, Demographic variables

## Abstract

Video gaming has become a popular leisure activity in many parts of the world, and an increasing number of empirical studies examine the small minority that appears to develop problems as a result of excessive gaming. This study investigated prevalence rates and predictors of video game addiction in a sample of gamers, randomly selected from the National Population Registry of Norway (*N* = 3389). Results showed there were 1.4 % addicted gamers, 7.3 % problem gamers, 3.9 % engaged gamers, and 87.4 % normal gamers. Gender (being male) and age group (being young) were positively associated with addicted-, problem-, and engaged gamers. Place of birth (Africa, Asia, South- and Middle America) were positively associated with addicted- and problem gamers. Video game addiction was negatively associated with conscientiousness and positively associated with neuroticism. Poor psychosomatic health was positively associated with problem- and engaged gaming. These factors provide insight into the field of video game addiction, and may help to provide guidance as to how individuals that are at risk of becoming addicted gamers can be identified.

Video gaming is one of the most popular contemporary recreational activities. It has been shown that 59 % of all Americans play video games (Ipsos MediaCT 2014). An average of 48 % of Europeans have played video games (Ipsos MediaCT 2012), and that 56 % of young adult Norwegians (aged 16–40 years) play video games regularly (Mentzoni et al. 2011). Among adolescents, the proportion of players is even higher, as demonstrated in a survey showing that 97 % of Americans aged 12–17 years play video games (Lenhart et al. 2008).

As video game playing has increased, so to have reports of problematic playing. The terms used to describe problematic video game play vary across the research literature (Brunborg et al. 2013). In the present study *video game addiction* is used as the preferred term and will be used to refer to problematic or pathological use of video games, where gaming leads to a functional impairment in daily life. Lemmens et al. (2009) define video game addiction as an “excessive and compulsive use of computer or video games that results in social and/or emotional problems; despite these problems, the gamer is unable to control this excessive use.” (Lemmens et al. 2009, p. 78).

Given that previous studies have used different assessment instruments and included diverse participant groups, prevalence rates for video game addiction vary across studies (Ferguson et al. 2011). In a literature review, Ferguson et al. (2011) found a prevalence rate of about 6.0 % for video game addiction. When excluding those that could rather been categorized as engaged gamers, the prevalence dropped to 3.1 %.

Employing this latter approach to classifying video game addiction, in which scale items reflecting salience, tolerance, and mood modification were regarded as indicators of engagement rather than addiction, Brunborg et al. (2013) found a prevalence of 4.2 % of addicted gamers, 12.9 % of problem gamers, 4.9 % of engaged gamers, and 78% of non-problem gamers among Norwegian adolescents. In contrast, using the original scoring criteria for the Game Addiction Scale for Adolescents (GASA; Lemmens et al. 2009), Mentzoni et al. (2011) estimated the prevalence rates in a representative sample of Norwegians aged 16–40 years old to be 0.6 and 4.1 % for video game addiction and problematic video gaming, respectively. The GASA is based on adapted DSM-IV criteria for pathological gambling (King et al. 2013), and hence, the Mentzoni et al. (2011) study might overestimate the prevalence rates, because an inclusion of Charlton’s (2002) engagement criteria would identify a number of gamers as addicted when they might not be.

Studies generally agree that males report more problems related to video gaming compared to females (Brunborg et al. 2013; Ferguson et al. 2011; Mentzoni et al. 2011). Concerning age, one study found that young age was a strong predictor for problematic use of video games (Mentzoni et al. 2011). Because (i) most of the research on video games is conducted on adolescents and teenagers (Williams et al. 2009) and/or samples of gamers (Pontes and Griffiths 2014), and (ii) there is paucity of studies based on general population samples (Wenzel et al. 2009), more research is needed to identify sociodemographic factors relevant for the risk of developing video game addiction.

As to the importance of other demographic variables, the research literature is relatively scarce. In relation to marital status, one study reported that the typical addicted gamer was single (Wenzel et al. 2009), while another study found video game addiction to be independent of educational background (Rehbein et al. 2010). Furthermore, it has been shown that unemployment may be a risk factor (Elliot et al. 2012), and is associated with high scores on video game addiction scales (Kim et al. 2008).

To the best of the authors’ knowledge, there are no studies investigating the relationship between video game addiction and country of origin in national population-based studies. Thus this issue should be explored further. A summary of recent prevalence studies found that there was a higher prevalence of problematic video gaming in East Asian populations, as compared to Western European, North American and Australian populations (King et al. 2012). Migration has been suggested to have a stress-inducing effect that can lead to mental illness (Bhugra and Jones 2001), but the picture is mixed and an effect of immigrant robustness has also been found, where immigrants are protected against mental health problems (Algeria et al. 2008). Case studies have reported that moving country can be a factor in excessive online gaming as a way of overcoming loneliness (Griffiths 2000).

Personality traits based on the Five-Factor Model (Costa and McCrae 1992) have previously been linked with different behavioral addictions (Andreassen et al. 2013). The five-factor model differentiates between five main dimensions: (1) Neuroticism (e.g., being nervous and anxiety prone), (2) Extroversion (e.g., being talkative and outgoing), (3) Openness to experience (being imaginative and intellectually oriented), (4) Agreeableness (e.g., being sympathetic and warm) and (5) Conscientiousness (e.g., being organized and prompt) (Wiggins 1996).

Previous studies have shown that video game addiction is positively correlated with neuroticism, and negatively with extraversion, agreeableness (Peters and Malesky 2008) and conscientiousness (Peters and Malesky 2008; Andreassen et al. 2013). These previous studies found no association in regards to openness. Since research in this area still is limited, more research is needed. The present study provides insight into the degree to which personality traits can explain video game related behavior. Furthermore, the present study provides new insight into the different personality profiles of different groups of video gamers.

A number of negative psychological health consequences have been reported in association to video game addiction (Choo et al. 2010), such as depression (Mentzoni et al. 2011; Van Roji et al. 2010), suicidal ideation (Wenzel et al. 2009; Rehbein et al. 2010) and anxiety (Wenzel et al. 2009; Rehbein et al. 2010). In addition, one study found that video game addicted boys had higher levels of sleep disturbance (Rehbein et al. 2010). Furthermore, Brunborg and colleagues (2013) reported that youth who were problem- or addicted gamers had a greater risk of feeling low, irritable or in a bad mood, nervous, being tired and exhausted, and feeling afraid, when compared to non-problem gamers. However, highly engaged gamers, who had comparable amounts of gaming time but who did not endorse the core addiction criteria, did not display greater risk for any of these health complaints.

Although several studies have been conducted on the relationship between health and video game addiction, few studies have used nationally representative samples of gamers. Since the present study uses a nationally representative sample it is a contribution to this gap in the research literature. Furthermore, as there are few studies investigating health in relation to different groups of gamers, the present study will also add to this literature in this regard.

The first aim of the present study was to estimate the prevalence rates of normal-, engaged-, problem-, and addicted gamers in a national representative population of gamers. The second aim was to investigate how strongly demographic factors, personality traits, and psychosomatic health are associated with the different gaming categories.

## Method

### Participants

Participants were randomly selected from the National Population Registry of Norway. The gross sample consisted of 24,000 people. They received a questionnaire assessing demography, video game addiction, personality factors, and health variables. Up to two reminders were sent to those who did not respond. A total of 875 questionnaires were returned due to various reasons (e.g., wrong addresses, deceased participants, being too ill to answer, being abroad at the time of the study or not understanding Norwegian). Thus, a total of 10,081 valid answers were received, resulting in a response rate of 43.6 %. A subset of 3389 respondents, aged 16–74 years (1351 females, mean age = 32.6 years) reported playing video games during the last 6 months.

Prevalence rates of different categories of gamers (addicted-, problem-, engaged- and normal gamer) were calculated in four different ways. Two different samples were used, one including all participants (*N* = 10,081) and one including active gamers only. Furthermore, prevalence rates are reported using Charlton’s (2002) division into core- and peripheral addiction criteria, and the original unidimensional scale scoring approach as described by Lemmens et al. (2009). All prevalence rates reported are weighted using inverse probability weights.

### Procedure

The study was based at the University of Bergen and carried out on behalf of the Norwegian Gambling and Foundation Authority during the autumn of 2013. All participants received the questionnaire by post. Participants were informed that responses would be treated confidentially, and that information about responders would be securely stored. Those who answered the questionnaire were offered the chance to be entered into a raffle for a gift voucher worth 500 Norwegian Kroner. The study was approved by the Regional Committee for Medical and Health Related Research Ethics in Western Norway (no. 2013/120).

### Instruments

General questions about participants’ background included gender, age, marital status (married/cohabiting or single/separated/divorced/widowed/widower), number of children they had care responsibilities for (from zero to five or more), highest completed education (from not ended primary school to completed PhD), personal income before taxes in the last year in bulks of 100 000 NOK (from 99,000 to 1,000,000 or more), employment status (full time employed, part time employed, student, homemaker, disabled/receiving social security or retired), and birthplace (Norway, countries in the Nordic region but outside of Norway, countries in Europe, Africa, Asia, North America, South America, Central America, or Oceania).

Personality traits were assessed using the Mini International Item Pool (Mini-IPIP; Donellan et al. 2006). Mini-IPIP is based on the five-factor model of personality and comprises 20 items where each personality trait consists of four items. Included dimensions are: 1) Neuroticism; 2) Extroversion; 3) Intellect/Imagination; 4) Agreeableness; and 5) Conscientiousness. Each item was answered on a five-point Likert scale (1 = *strongly disagree* to 5 = *strongly agree*). Internal consistency (Cronbach’s alpha) for the scale in the current study was 0.80 for extraversion, 0.75 for agreeableness, 0.68 for conscientiousness, 0.70 for neuroticism, and 0.66 for intellect/imagination (*n* = 3622).

An eight-item scale to assess psychosomatic health symptoms were constructed (headache, shoulder-/neck pain, stomach-/intestinal pain, sleep problems, feeling sad/depressed, feeling restless and nervous, feeling tired or sleepy during daytime, and heart palpitations) based on previous scales developed for psychosomatic symptoms (Eriksen et al. 1999; Hagquist 2008; Kroenke et al. 2002; Takata and Sakata 2004; Thorndike et al. 1952). Participants were asked to consider how often they had experienced these symptoms during the past 2 months choosing from the following options: “never”, “less than once a month”, “1–3 times a month”, “1–2 times a week”, and “3 times a week or more often”. Internal consistency (Cronbach’s alpha) for the scale was 0.83 (*n* = 3622). A total score of all eight items was divided by eight and used in the analysis.

The seven-item version of the Game Addiction Scale for Adolescents (GASA; Lemmens et al. 2009) was used to assess game addiction. Respondents indicated their responses on a five-point scale (1 = *never* to 5 = *very often*). Internal consistency (Cronbach’s alpha) for the scale was 0.84 (*n* = 3622).

The respondents were categorized into four different categories of gamers, namely addicted gamer, problem gamer, engaged gamer and normal gamer (Brunborg et al. 2013, 2015). Respondents who indicated that all four items measuring the core components of addiction (relapse, withdrawal, conflict and problems) had occurred at least “sometimes” (3) were classified as addicted to video games. Respondents scoring at least “sometimes” (3) on two or three of the same items were classified as problem gamers. Respondents scoring at least 3 on the three first items (salience, tolerance, mood modification) but who did not score 3 or above on more than one of the core criteria were classified as engaged. The remaining respondents were categorized as non-problem gamers.

The demographic variables were recoded in the following manner: gender was dichotomized (1 = *female* and 2 = *male*), three age groups were constructed (1 = *51*–*74*, 2 = *31*–*50* and 3 = *16*–*30*), marital status was dichotomized (1 = *living with a partner* and 2 = *living alone*), place of birth was categorized into three groups (1 = *Africa, Asia, South and Middle America*, 2 = *Europe, North-America, Oceania* and 3 = *Norway*), level of education was categorized into three groups (1 = *lower- or upper secondary education,* 2 = *upper secondary vocational education* and 3 *= higher education*), employment status was dichotomized (1 = *unemployed* and 2 = *employed*).

For the personality traits and psychosomatic health measure, median split was used to dichotomize both parameters, creating groups scoring above (1) and below (2) the median for the personality traits, and creating groups scoring above (2) and below (1) the median for psychosomatic health.

### Statistics

Descriptive statistics of nominal variables were calculated in terms of distribution. Pearson product–moment correlation coefficients were calculated in order to investigate the interrelationship between the predictor variables in the study. Using the sample that reported playing video games during the last 6 months, crude and adjusted multinomial regression analyses were conducted with the categorical video gaming variable (“addicted gamer”, “problem gamer”, “engaged gamer” and “normal gamer”) as the dependent variable. “Normal gamer” was used as the reference category. Gender, age, place of birth, marital status, education level and employment status were entered in step one, personality was included in step two, and psychosomatic health were entered in step three. The prerequisites for conducting this type of analysis were fulfilled. The statistical analyses were conducted using IBM SPSS Statistics, version 22.

## Results

Table 1 shows descriptive data for the sample. The percentage of males reporting playing video games the last 6 months were 62.7 and 37.3 % were females (*N* = 3389). Table 2 shows prevalence (weighted) rates for the video game sample, and the whole population sample, using Charlton’s core and peripheral factor’s solution. The prevalence estimate for video game addiction was 1.41 % (CI = 1.03, 1.80) in the video game sample, and 0.53 % (CI = 0.39, 0.67) for the whole population sample.

**Table 1:** Descriptive data for the sample (*N* = 3389)

		Percentage	Mean (SD)
Gender	Male	62.7	
	Female	37.3	
Age group	16–30 years	49.7	
	31–50 years	40.8	
	51–74 years	9.5	
Marital status	Married, living with a partner	55.7	
	Single, separated, divorced, widow, widower	44.3	
Place of birth	Norway	88.7	
	Europe, North America, Oceania	8.1	
	Africa, Asia, South and Middle America	3.2	
Level of education	Higher education	42.4	
	Upper secondary vocational education	15.7	
	Lower - or upper secondary education	41.9	
Employment status	Full-time, part-time, student	87.8	
	Not working	12.2	
Personality	Extraversion		1.42 (0.49)
	Agreeableness		1.44 (0.50)
	Consentiousness		1.51 (0.50)
	Intellect/Imagination		1.34 (0.47)
	Neuroticism		1.47 (0.50)
Psychosomatic health			1.54 (0.50)

**Table 2:** Prevalence (weighted) rates for the different groups of gamers in a population of gamers and in the population as a whole

	Video game sample (*n* = 3035) [95 % CI]	Whole population sample (*N* = 10 081) [95 % CI]
Addicted gamers	1.41 % [1.03, 1.80]	0.53 % [0.39, 0.67]
Problem gamers	7.30 % [6.45, 8.15]	2.73 % [2.41, 3.05]
Engaged gamers	3.88 % [3.25, 4.51]	1.42 % [1.19, 1.66]
Normal gamers	87.4 % [86.3, 88.5]	95.3 % [94.9, 95.7]

Table 3 shows prevalence (weighted) rates for the video game sample, and the whole population sample, after Lemmens original scoring. The prevalence estimate for video game addiction was 0.89 % (CI = 0.58, 1.19) in the video game sample, and 0.33 % (CI = 0.21, 0.44) for the whole population sample.

**Table 3:** Prevalence (weighted) rates for the different groups of gamers in a population of gamers and in the population as a whole, after Lemmens original scoring

	Video game sample (*n* = 3035) [95 % CI]	Whole population sample (*N* = 10 081) [95 % CI]
Addicted gamers	0.89 % [0.58, 1.19]	0.33 % [0.21, 0.44]
Problem gamers	8.07 % [7.18, 8.96]	3.00 % [2.66, 3.34]
Normal gamers	91,0 % [90.1, 92.0]	96.7 % [96.3, 97.0]

Table 4 shows the correlations between all predictor variables in the study. The strongest correlations was between age and level of education (*r* = 0.35), marital status and education (*r* = 0.38), and older age group and marital status (*r* = 0.38).

**Table 4:** Correlation coefficients (Pearson correlation) and Phi coefficients between all study variables (gender, age group, marital status, place of birth, level of education, employment status, personality [extraversion, agreeableness, conscientiousness, intellect/imagination, neuroticism] and health)

		1	2	3	4	5	6	7	8	9	10	11	12
1.	Gender (a)	1	0.01	0.05**	−0.03	0.00	−0.08**	0.04*	0.24**	0.07**	−0.06**	0.22**	−20**
2.	Age group (b)		1	0.42**	−0.01	0.35**	−0.13**	−0.02	−0.03*	0.11**	−0.07**	−0.03*	0.10**
3.	Marital status (c)			1	−0.07**	0.38**	0.07**	0.05**	0.02	0.13**	−0.07**	−0.04*	0.08**
4.	Place of birth (d)				1	−0.05**	0.03	0.02	0.07**	0.01	−0.01	−0.11**	0.00
5.	Level of education (e)					1	0.13**	0.03	0.06**	0.11**	0.04*	0.08**	0.05**
6.	Employment status (f)						1	0.07**	0.04*	0.06**	0.03	−0.11**	0.08**
7.	Extraversion							1	0.24**	0.10**	0.17**	−0.11**	0.10**
8.	Agreeableness								1	0.14**	0.14**	−0.05**	−0.01
9.	Conscientiousness									1	0.01	−0.10**	0.15**
10.	Intellect/Imagination										1	−0.07**	−0.02
11.	Neuroticism											1	−0.31**
12.	Psychosomatic health												1

*p < .05. **p < .01

(a) 1 = Female, 2 = Male

(b) 1 = 51–74 years, 2 = 31–50 years, 3 = 16–30 years

(c) 1 = Living with a partner, 2 = Not living with a partner

(d) 1 = Norway, 2 = Europe, North America or Oceania 3 = Africa, Asia or South/Middle America

(e) 1 = Higher education, 2 = Vocational education, 3 = Elementary school or high school

(f) 1 = Working (full time or part time) or student, 2 = Not working

Table 5 presents the results from the univariate (crude) multinomial logistic regression analysis in terms of odds ratio (OR) and 95 % confidence intervals (95 % CI).

**Table 5:** Multinominal logistic regression analysis (crude) where video game addiction (1 = addicted gamer, 2 = problem gamer, 3 = engaged gamer, 4 = normal gamer) comprised the dependent variable, for which normal gamer comprises the reference category

		Crude OR [95 % CI]		
		Addicted	Problem	Engaged
Gender	Male	1.00	1.00	1.00
	Female	0.50 [0.26, 0.96]*	0.51 [0.38, 0.68]**	0.62 [0.43, 0.9]*
Age Group	16–30 years	1.00	1.00	1.00
	31–50 years	0.20 [0.09, 0.43]**	0.48 [0.36, 0.63]**	0.44 [0.30, 0.64]**
	51–74 years	0.26 [0.07, 0.97]*	0.15 [0.06, 0.35]**	0.27 [0.11, 0.63]**
Marital status	Single, separated, divorced, widow, widower	1.00	1.00	1.00
	Married, living with a partner	0.21 [0.10, 0.42]**	0.55 [0.43, 0.71]**	0.35 [0.24, 0.50]**
Place of birth	Norway	1.00	1.00	1.00
	Europe, North-America, Oceania	0.48 [0.11, 2.10]	1.04 [0.65, 1.67]	0.77 [0.39, 1.56]
	Africa, Asia, South- and Middle America	5.80 [2.56, 13.16]**	2.92 [1.72, 4.94]**	1.49 [0.61, 3.63]
Level of education	Higher education	1.00	1.00	1.00
	Upper secondary vocational education	1.74 [0.71, 4.26]	2.29 [1.54, 3.41]**	1.58 [0.90, 2.78]
	Lower - or upper secondary education	2.50 [1.29, 4.86]**	3.09 [2.27, 4.21]**	2.80 [1.88, 4.17]**
Employment status	Full-time, part-time, student	1.00	1.00	1.00
	Not working	1.63 [0.78, 3.39]	1.60 [1.13, 2.25]**	1.92 [1.24, 2.97]**
Personality	Extraversion	0.48 [0.27, 0.85]*	0.79 [0.61, 1.01]	0.69 [0.49, 0.97]*
	Agreeableness	0.43 [0.24, 0.77]**	0.60 [0.47, 0.78]**	0.60 [0.43, 0.84]**
	Conscientiousness	0.21 [0.10, 0.42]**	0.29 [0.21, 0.39]**	0.38 [0.26, 0.55]**
	Neuroticism	2.46 [1.30, 4.64]**	1.92 [1.46, 2.51]**	1.52 [1.07, 2.16]*
	Intellect/Imagination	1.25 [0.68, 2.29]	1.41 [1.06, 1.88]*	1.34 [0.91, 1.95]
Psychosomatic health	High score	1.00	1.00	1.00
	Low score	0.43 [0.23, 0.79]**	0.29 [0.21, 0.39]**	0.43 [0.30, 0.63]**

*OR* Odds ratio, *CI* Confidence interval

*p < .05. **p < .01

Table 6 presents the data from the adjusted multinomial regression analysis.

**Table 6:** Multiple regression analysis (adjusted), where video game addiction (1 = addicted gamer, 2 = problem gamer, 3 = engaged gamer, 4 = normal gamer) comprised the dependent variable, for which normal gamer comprises the reference category

		Adjusted OR 1 [95 % CI]			Adjusted OR 2 [95 % CI]			Adjusted OR 3 [95 % CI]		
		Addicted	Problem	Engaged	Addicted	Problem	Engaged	Addicted	Problem	Engaged
Gender	Male	1.00	1.00	1.00	1.00	1.00	1.00	1.00	1.00	1.00
	Female	0.39 [0.18, 0.82]*	0.48 [0.35, 0.66]**	0.60 [0.41, 0.90]*	0.37 [0.17, 0.81]*	0.51 [0.36, 0.72]**	0.60 [0.38, 0.93]*	0.35 [0.16, 0.77]**	0.42 [0.29, 0.60]**	0.48 [0.3, 0.76]**
Age Group	16–30 years	1.00	1.00	1.00	1.00	1.00	1.00	1.00	1.00	1.00
	31–50 years	0.39 [0.16, 0.95]*	0.58 [0.41, 0.81]**	0.73 [0.46, 1.15]	0.37 [0.15, 0.91]*	0.63 [0.44, 0.91]*	0.71 [0.44, 1.14]	0.35 [0.14, 0.87]*	0.60 [0.42, 0.86]**	0.71 [0.44, 1.14]
	51–74 years	0.46 [0.11, 1.88]	0.17 [0.07, 0.41]**	0.38 [0.15, 0.94]*	0.52 [0.13, 2.17]	0.2 [0.08, 0.52]**	0.32 [0.11, 0.92]*	0.25 [0.03, 1.88]	0.24 [0.90, 0.63]**	0.38 [0.13, 1.09]
Marital status	Single, separated, divorced, widow, widower	1.00	1.00	1.00	1.00	1.00	1.00	1.00	1.00	1.00
	Married, living with a partner	0.28 [0.12, 0.63]**	1.02 [0.74, 1.40]	0.58 [0.37, 0.89]*	0.35 [0.15, 0.79]*	1.04 [0.74, 1.46]	0.68 [0.43, 1.06]	0.39 [0.17, 0.86]*	1.07 [0.76, 1.50]	0.71 [0.45, 1.10]
Place of birth	Norway	1.00	1.00	1.00	1.00	1.00	1.00	1.00	1.00	1.00
	Europe, North-America, Oceania	0.83 [0.19, 3.68]	1.40 [0.85, 2.31]	0.95 [0.44, 2.05]	0.70 [0.16, 3.15]	1.07 [0.62, 1.82]	0.90 [0.41, 1.95]	0.71 [0.16, 3.23]	1.00 [0.57, 1.75]	0.73 [0.31, 1.71]
	Africa, Asia, South and Middle America	4.91 [1.85, 13.03]**	3.00 [1.70, 5.28]**	1.62 [0.65, 4.00]	4.45 [1.62, 12.24]**	2.58 [1.40, 4.77]**	1.69 [0.68, 4.22]	4.91 [1.77, 13.64]**	3.08 [1.65, 5.78]**	1.88 [0.75, 4.72]
Level of education	Higher education	1.00	1.00	1.00	1.00	1.00	1.00	1.00	1.00	1.00
	Upper secondary vocational education	1.24 [0.49, 3.11]	2.05 [1.36, 3.09]**	1.32 [0.73, 2.38]	0.94 [0.34, 2.58]	1.76 [1.14, 2.71]*	1.30 [0.71, 2.38]	0.96 [0.35, 2.66]	1.86 [1.19, 2.90]**	1.33 [0.73, 2.44]
	Lower - or upper secondary education	1.00 [0.48, 2.11]	2.14 [1.49, 3.07]**	1.83 [1.15, 2.91]**	0.99 [0.46, 2.12]	2.01 [1.38, 2.92]**	1.78 [1.1, 2.88]*	0.95 [0.44, 2.03]	2.01 [1.37, 2.94]**	1.81 [1.12, 2.92]*
Employment status	Full-time, part-time, student	1.00	1.00	1.00	1.00	1.00	1.00	1.00	1.00	1.00
	Not working	1.93 [0.88, 4.24]	1.81 [1.23, 2.66]**	1.99 [1.25, 3.19]**	1.55 [0.70, 3.46]	1.27 [0.83, 1.94]	1.66 [1.01, 2.75]*	1.43 [0.61, 3.31]	1.25 [0.82, 1.92]	1.63 [0.96, 2.69]
Personality	Extraversion				0.58 [0.30, 1.10]	0.99 [0.74, 1.34]	0.74 [0.51, 1.09]	0.62 [0.32, 1.18]	1.06 [0.78, 1.44]	0.81 [0.55, 1.20]
	Agreeableness				0.81 [0.42, 1.54]	0.72 [0.53, 0.98]*	0.76 [0.52, 1.13]	0.83 [0.43, 1.61]	0.70 [0.52, 0.97]*	0.78 [0.52, 1.15]
	Conscientiousness				0.31 [0.15, 0.66]**	0.39 [0.24, 0.47]**	0.55 [0.37, 0.82]**	0.33 [0.16, 0.71]**	0.37 [0.26, 0.51]**	0.60 [0.40, 0.90]*
	Neuroticism				2.48 [1.23, 5.02]*	1.74 [1.23, 2.38]**	1.35 [0.91, 2.00]	2.21 [1.08, 4.55]*	1.49 [1.08, 2.06]*	1.21 [0.81, 1.82]
	Intellect/Imagination				1.70 [0.84, 3.45]	1.51 [1.09, 2.08]*	1.49 [0.98, 2.27]	1.59 [0.78, 3.23]	1.48 [1.06, 2.06]*	1.41 [0.92, 2.14]
Psychosomatic health	High score							1.00	1.00	1.00
	Low score							0.64 [0.32, 1.27]	0.33 (0.23, 0.47]**	0.46 (0.30, 0.70]**

*OR* Odds ratio, *CI* Confidence interval

*p < .05. **p < .01

In both the crude and adjusted analyses, being addicted-, problem- or engaged gamer was significantly and negatively associated with gender, indicating that male respondents were more likely than female respondents to belong to all of these categories.

Being 31–50 years old was significantly and negatively associated with being an addicted- or problem gamer compared to the contrast group (16–30 years) in both the crude and adjusted analysis. Being 51–80 years was negatively associated with being an addicted gamer, problem gamer or an engaged gamer compared to the contrast group in the crude analysis. The effect was still significant when adjusting for personality traits, but the association with being an engaged gamer did not remain significant when adjusting for psychosomatic health.

Being born in Africa, Asia, South- or Middle America was positively and significantly related to being an addicted- or problem gamer in both the crude and adjusted analysis. In the crude analysis, a high score on extraversion was significantly and negatively associated with being an addicted- or engaged gamer compared to those having a low score. In the adjusted analysis, none of the associations remained significant. In the crude analysis, agreeableness was significantly and negatively associated with being an addicted-, problem-, or engaged gamer. In the adjusted analysis only, the negative association with being a problem gamer remained. For conscientiousness there was a significant and negative association with being an addicted-, problem- or engaged gamer both in the crude- and adjusted analyses. In the crude analysis, neuroticism was positively and significantly associated with being an addicted-, problem- or engaged gamer. However, in the adjusted model, the association with being an engaged gamer did not remain significant. In the crude and adjusted analysis, intellect/imagination was significantly and positively associated with being a problem gamer.

Having a low score on the psychosomatic health scale was negatively associated with being an addicted-, problem- or engaged gamer in the crude analysis. In the adjusted model, the association with being an addicted gamer did not remain significant.

The full model containing all predictors (adjusted analysis) was statistically significant (*χ*^*2*^ = 358.24, *df* = 45, *p* < .01). Furthermore, the model as a whole explained between 10.6 % (Cox and Snell R square) and 17.3 % (Nagelkerke R squared) of the variance in video game addiction and correctly classified 88.3 % of all the cases.

## Discussion

Using the whole sample, and applying the original scoring of GASA, both the prevalence of addicted gamers (0.33 %) and the prevalence of problem gamers (3.0 %), were lower than a previous Norwegian study (addicted gamers: 0.6 %, problem gamers: 4.1 %; see Mentzoni et al. 2011). Furthermore, the prevalence of addicted gamers was lower than what has been found worldwide (6.0 %, Ferguson et al. 2011). This could indicate that the prevalence of video game addiction is lower in Norway than worldwide, or it might reflect that the literature review by Ferguson et al. (2011) only included studies with youths and young adults.

In comparison, when using the sample of active video gamers and the interference approach, the prevalence numbers were higher for all groups of gamers: addicted (1.41 %), problem (7.3 %) and engaged (3.9 %). However, the prevalence of addicted gamers was lower than what has been found worldwide (Ferguson et al. 2011). Furthermore, when comparing these results with Brunborg et al. (2013), who used an adolescent population, the prevalence numbers reported here are lower for all categories of gamers. Thus, this latter finding supports the interpretation that the prevalence rates reported by Ferguson et al. (2011) were high because it included studies with youths and young adults only.

The results of the present study are in line with previous research stating that males report more problems with gaming than females (Brunborg et al. 2013; Ferguson et al. 2011; Mentzoni et al. 2011). Males were in the present study were 2.9 times more likely than females to belong to the addicted gamers category. Furthermore, there were no noteworthy changes when including personality traits and psychosomatic health in the analysis. This suggests that gender is independent of these variables. The results further support research suggesting that being single is positively associated with excessive video game use (Wenzel et al. 2009), and the literature suggesting that younger age is associated with problems with video game use (Mentzoni et al. 2011). Respondents in the youngest age group were more likely to belong to the addicted group than the middle age (2.9 times more likely) and the oldest age group (4 times more likely). Moreover, respondents in the youngest age group were more likely to belong to the group of problem gamers than the oldest age group (4.2 times more likely). However, it should be noted that gaming is a relatively new phenomenon hence cohort-effects may be at play. As the younger video gaming generation grows up, gaming will probably be more uniformly distributed across age groups.

Respondents that were born in Africa, Asia, South America or Central America were 4.9 times more likely to belong to the group of addicted gamers, and 3.1 times more likely to belong to the group of problem gamers, compared to respondents born in Norway. The present authors have not been able to identify previous research investigating video game addiction among immigrants. Previous findings are mixed as to whether immigrants are in the risk group for mental health problems in general (e.g., Bhugra and Jones 2001; Algeria et al. 2008). However, previous research has found that there is a higher prevalence of problematic video gaming in East Asian populations, as compared to Western European, North American and Australian populations (King et al. 2012), which might lend support to the idea that immigrants from this region might be more susceptible to develop video game addiction, because of their general interest for gaming, and not because of immigration. However, it could also be the case that gaming provides a social outlet for lonely and/or non-integrated individuals and that they may use online media as a way of forming friendships with other like-minded individuals (Cole and Griffiths 2007).

Video game addiction was independent of level of education, and is in accordance with previous research (Rehbein et al. 2010). However, the results of the present study suggest that problem- and engaged gamers have a lower degree of education. One could speculate that gamers with higher level of education would put more time and effort into their careers than gamers with a low education, and therefore spend less time gaming. A confounding variable in relation to this association might be young age, since the group of respondents with the lowest level of education will consist of both adults that have completed their degree of education and adolescents that are still studying. Such interpretation is partly supported by the results, where a moderate correlation between age and level of education was found.

Previous studies have found an association between unemployment and problematic video game and internet use (Elliot et al. 2012; Kim et al. 2008), but this association was not found in the present study in relation to video game addiction. The present results also support previous findings concerning personality and video game addiction with regard to neuroticism, conscientiousness and intellect/imagination (Peters and Malesky 2008; Andreassen et al. 2013). As people who are high on neuroticism can experience more anxiety and depression (Costa and McCrae 1992), they might use video game playing as an escape for their problems. Furthermore, being high on neuroticism is has been shown to be related to impulsiveness (Costa and McCrae 1992), that might make it easier to discard other activities in favor of playing video games. The results of the present study showed that respondents who scored high on conscientiousness were three times less likely to belong to the group of addicted gamers, and that conscientiousness was negatively associated with addicted-, problem-, or engaged gamers. A possible reason for this might be that people who score high on conscientiousness typically are dutiful and self-disciplined (Costa and McCrae 1992), traits that can be said to be incompatible with heavy video game playing.

In contrast to Peters and Malesky (2008), no significant relationship was found between extraversion or agreeableness and video game addiction. Because Peters and Malesky (2008) used a sample of gamers from a specific online game (i.e. World of Warcraft), the connection between video game addiction and extraversion or agreeableness may only be true for people who play this game, or similar types of games.

Unlike previous studies (Rehbein et al. 2010; Brunborg et al. 2013) the results of the current study indicate no association between video game addiction and poor psychosomatic health. However, an association between having a low score on psychosomatic health and being in the group of problem gamers or in the group of engaged gamers was found. The results indicate that the high scoring group on psychosomatic symptoms is three times more likely to belong to the group of problem gamers than the low scoring group. The reason why the results of the present study differ from previous findings might differences in the assessment of psychosomatic health. For instance, Brunborg et al. (2013) looked at particular factors of psychosomatic health, such as “feeling low”, “trouble sleeping” and “tired”, while the current study pooled several items together. In addition, the fact that the current study controlled for several demographic variables and personality factors might further explain why different results were found. The results support a distinction between different groups of gamers as the personality traits investigated show different associations within different groups of gamers. For example, the trait of neuroticism is only significant for addicted gamers and problem gamers, but not for engaged gamers.

By using a sample randomly selected from the national population registry, the results can be generalized across the video gaming population. There is a need for further population-based studies given the lack of such studies to date (Wenzel et al. 2009). Furthermore, most previous research has been conducted on adolescents and teenagers (Williams et al. 2009). The current study also obtained different prevalence rates by using different scoring methods. In this way, the study offers an opportunity to compare different prevalence numbers from previous studies.

One shortcoming of the present study is that it did not differentiate between different types of games. Studies have shown that characteristics of games can be of importance in the development of video game addiction (King et al. 2011). Several studies using specific games such as *Everquest* (Williams et al. 2008; Griffiths et al. 2004) have reported different results than the present study, and MMORPGs have for example been found to be more addictive than other games (Rehbein et al. 2010). More research is needed to clarify whether playing specific types of games is typical for gamers belonging to the four different groups of gamers. The results concerning place of birth could also have been different if more detailed response alternatives than continent had been used. The study also lacked a measure of how much respondents play. Due to cross-sectional design the present study is further limited, and we are precluded from drawing inferences causal relations between the variables. Further longitudinal studies are needed in order to conclude about directionality between variables. The study also suffers from the many biases known that utilize self-report data (e.g., recall biases, social desirability biases, etc.).

## Conclusions

The present study showed the prevalence of addicted gamers to be 1.4 %, problem gamers to be 7.3 % and engaged gamers to be 3.9 %. The results identified the following factors to be associated with video game addiction: being of male gender, being young in age, living alone, being born in Africa, Asia, South America or Central America, scoring low on conscientiousness, scoring high on neuroticism, and having poor psychosomatic health. These factors provide insight into the field of video game addiction, and may help to provide guidance as to how one can identify people that are in risk of becoming addicted gamers.
